# DMINDA: an integrated web server for DNA motif identification and analyses

**DOI:** 10.1093/nar/gku315

**Published:** 2014-04-21

**Authors:** Qin Ma, Hanyuan Zhang, Xizeng Mao, Chuan Zhou, Bingqiang Liu, Xin Chen, Ying Xu

**Affiliations:** 1 Computational Systems Biology Laboratory, Department of Biochemistry and Molecular Biology, and Institute of Bioinformatics, University of Georgia, Athens GA 30602, USA; 2 BioEnergy Science Center (BESC), Oak Ridge National Laboratory, Oak Ridge, Tennessee 37831, USA; 3 College of Computer Science and Technology, Jilin University, Changchun, China; 4 School of Mathematics, Shandong University, Jinan, Shandong, China

## Abstract

DMINDA (**D**NA **m**otif **i**dentification a**nd a**nalyses) is an integrated web server for DNA motif identification and analyses, which is accessible at http://csbl.bmb.uga.edu/DMINDA/. This web site is freely available to all users and there is no login requirement. This server provides a suite of *cis*-regulatory motif analysis functions on DNA sequences, which are important to elucidation of the mechanisms of transcriptional regulation: (i) *de novo* motif finding for a given set of promoter sequences along with statistical scores for the predicted motifs derived based on information extracted from a control set, (ii) scanning motif instances of a query motif in provided genomic sequences, (iii) motif comparison and clustering of identified motifs, and (iv) co-occurrence analyses of query motifs in given promoter sequences. The server is powered by a backend computer cluster with over 150 computing nodes, and is particularly useful for motif prediction and analyses in prokaryotic genomes. We believe that DMINDA, as a new and comprehensive web server for *cis*-regulatory motif finding and analyses, will benefit the genomic research community in general and prokaryotic genome researchers in particular.

## INTRODUCTION

DNA *cis*-regulatory elements, or ‘motif’ for short, contain the transcription factor (TF) binding sites and other conserved functional elements in the promoter regions of genes. Such motifs are usually short (8–12 bp) and tend to be conserved at the sequence level to facilitate specific binding with their *trans* regulators (1). Three types of computational problems have been formulated and extensively studied associated with the motif-finding problem: (i) *de novo* motif finding (2), (ii) scanning for motif instances of a query motif in specified promoter regions (3) and (iii) motif comparison, typically needed for prediction-reliability assessment (4). Although these problems have been extensively studied and a few hundred thousand articles have been published since mid-80s, new papers still come out with relatively high rates, indicating that these problems remain to be important and unsolved. 

A number of web servers for motif finding have been deployed in the public domain, including YMF (5), SCOPE (6), RBPmotif (7), DRIMUST (8), MEME suite (9) and GIBBs (10), among which the MEME remains the most popular in the past two decades. Most of these programs are designed for motif identification where associated motif analyses, such as those defined above, tend to be treated as different problems and hence not included in these servers. Interestingly, while these motif-finding servers are all applicable to motif identification in prokaryotes, none of them are designed to take full advantage of the special characteristics of prokaryotic genomes to make motif identification more reliable. One such characteristic is that transcriptionally co-regulated genes may group into ‘operons’ (11,12) and share common promoters. It is worth noting that operons can be computationally predicted using only sequence information that is independent of the information typically used for motif predictions (13), hence providing complementary information for motif identification. We have recently developed an integrated toolkit for motif identification and analyses, called BoBro (14,15), and implemented it as a standalone command-line software package. It can reliably identify statistically significant motifs at a genome scale on both the *Escherichia coli* K12 and the human genomes, as have been demonstrated on large test sets, particularly on noisy data (15), and does so more efficiently and accurately than the best available tools such as MEME. In addition, its motif-scanning and comparison capabilities have also been demonstrated to be highly reliable (15). Since its publication in April of 2013, this standalone software has been downloaded 1964 times. Here, we present a web server, DMINDA (DNA motif identification and analyses), for this toolkit with a few new features to facilitate a wider range of applications.

Key features of the server include (i) a high-performance web service for motif prediction and analyses, powered by a computer cluster with 150 computing nodes; (ii) identification and evaluation of conserved motifs at a genome scale (for prokaryotes) along with estimated statistical significance scores; (iii) an operon database DOOR (12), in support of prokaryotic motif identification in particular; (iv) accurate scan for all instances of a query motif in specified genomic sequences along with estimated statistical significance scores; (v) motif comparison and clustering for identified motifs, which takes into consideration the weakly conserved signals in the flanking regions of the motifs; and (vi) correlational analyses among the identified motifs to facilitate inference of joint regulatory relationships among TFs. We believe that this server will provide a highly useful and easy-to-use platform for motif identification and analyses complementary to the existing tools in the public domain.

## DMINDA WEB SERVER

### Basic information

**Figure 1.**
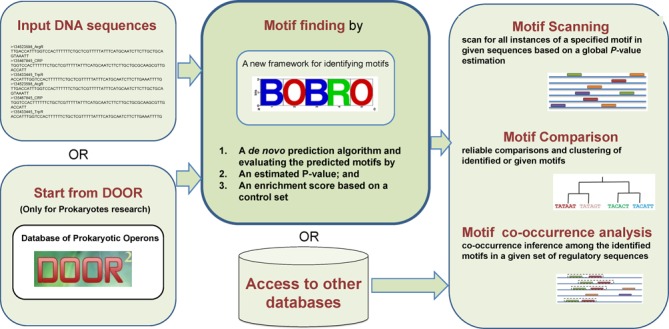
Four motif analysis functionalities are accessible by the following clickable buttons on the front page of DMINDA: Motif finding, Motif scanning, Motif comparison and Motif co-occurrence analysis. And 21 motif databases are integrated in Access to other databases.

DMINDA has a number of capabilities in support of motif finding and analyses: (i) a *de novo* motif-finding algorithm along with motif assessment based on information extracted from a control set; (ii) scanning motif instances of a query motif along with estimated *P*-value; (iii) motif comparison and clustering; and (iv) co-occurrence analyses of query motifs in specified promoter regions, each of which is up to 300 bp long. These functionalities can be accessed through clickable menus or buttons, namely, Motif finding, Motif scanning, Motif comparison, and Motif co-occurrence analysis, on the front page of DMINDA (see Figure 1 and Supplementary Tutorial S1). All the source codes for performing these functionalities along with their documentations are available at http://csbl.bmb.uga.edu/DMINDA/download.php. In addition, the server has an Access to other databases page through which a user can retrieve needed motif information from 21 DNA-motif databases (16–35) (Supplementary Table S1) and carry out relevant analyses functions in specified DNA sequences.

For each of these functionalities, the server prompts the user with an input page for information collection. For missing items that are needed for each application, the server will automatically prompt the user to include them until no required information is missing. For a submitted job, the server will generate a unique job ID for the result retrieval when it is done, where the ID along with some additional information will be sent to the user if an email address is provided during job submission. Specifically, each ID has a suffix indicating the corresponding analysis functionality, with *f*, *s*, *c* and *a* representing motif finding, scanning, comparison and clustering, and co-occurrence analysis, respectively. The result will be displayed on a designated page and saved in our local database for six months, which is secure and only accessible through the corresponding job ID.

In terms of the actual computing time, DMINDA can predict motifs for a whole prokaryotic genome (e.g. with 2000 promoters) in several hours using the backend computing power for the server, and is capable for motif scanning at a larger scale, e.g. for the human genome (with more than 20 000 promoters). More detailed computing related information about each of the four main prediction capabilities are shown in Supplementary Table S2, along with detailed running time for large-scale jobs. The following summarizes each key functionality of the server.

### Motif identification and evaluation

This function identifies a set of statistically significant motifs (if any) in a set of provided promoter sequences. The underlying algorithm (14) finds conserved motifs in the provided sequences through formulating and solving the problem as a combinatorial optimization problem (Supplementary Method S1). For each predicted motif, the algorithm calculates statistical significances as follows (15). We use **P** to denote the provided promoters and **G** a control set, which could be an entire genome or randomly generated sequences. Each motif is evaluated using (i) a *P*-value with respect to a null hypothesis that it appears in **P** by chance so the smaller a *P*-value, the less likely the motif is found by chance and hence statistically more significant; along with (ii) an enrichment score of the motif occurring in **P** against in **G** so that the higher the score, the more enriched the motif is in **P** than in the general background **G**. Here, a predicted motif is considered as significant if its *P*-value is lower than 1e-2 and the enrichment score is larger than two (if available).

#### Input and analysis

The input to this functionality is a set of promoter sequences in the FASTA format. The server allows a user to include a set of control sequences (also in FASTA format) so that the predicted motifs should be statistically significant in the given promoter sequences but not in the control set. The calculation of this functionality is done by BoBro (14,15) with the following three parameters: the number, the minimal and maximal lengths of the to-be-identified motifs. A number of input example files are given in Supplementary Tutorials S2 and S3.

#### Main and the result pages for motif identification

The main page for motif identification has the following functional menus/buttons (Supplementary Tutorial S2): (i) the Input query sequences page prompts the user to enter the query sequences by pasting them in the input box or by submitting an input file from the local computer. **Sample** in this menu is used to upload a set of sample sequences to the input box and **Select from DOOR** automatically extracts the promoter sequences from a specified prokaryotic genome in the DOOR database, which now has 2072 prokaryotic genome sequences (see an example in a later section); (ii) **Include control sequences** prompts the user to include control sequences if choosing to do so. The **Sample** button within this menu uploads a set of randomly generated control sequences. If the user wants to select an entire genome as the control for calculating an enrichment score, he/she needs to select a specific genome after clicking on **Select from DOOR** (see an example in a later section); (iii) **Set parameters** allows the user to adjust the three parameters (defined above) of the motif-identification program, which otherwise use the default parameters; (iv) **Submit job** allows the user to submit a job, where it is optional for the user to leave an email address for informing the user that a job is done, with a clickable link where the computing result can be retrieved, otherwise a result page will pop up when a job is done if the user stays on the job submission page waiting for the job to be done. The **Submit job** menu has the same functions across all the other motif analysis functionalities in the following subsections.

**Figure 2.**
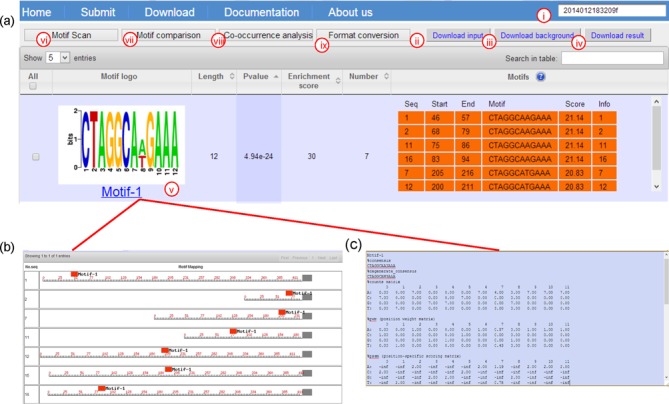
Result page of *de novo* motif finding. (**a**) Predicted motifs with nine functional buttons, where **i** is a searching box showing corresponding job ID, and a user can download the submitted query, control sequences and the predictions by clicking **ii**, **iii** and **iv**, respectively, and the other relevant details can be accessed by clicking button **v**. **vi–viii** allow a user to do three follow-up motif analysis functions and **ix** provides a format conversion capability to inter-convert file formats used in our server, MEME and the Uniprobe database. (**b**) The locational information of the predicted motif instances of a motif compared to downstream genes. (**c**) The detailed information of a predicted motif, including consensus, motif counts matrix, PWM, PSSM, information content and this motif in other formats, e.g. MEME and Uniprobe.

A result page lists all the identified motifs (if any) for the given query sequences, with each row representing one motif showing the following information: motif logo, motif length, the *P*-value, the enrichment score, the number of instances, the genomic location for each identified instance in the query sequences, the sequence alignment of the motif, and a clickable link to the position weight matrix, position-specific scoring matrix and a graphical mapping of predicted instances in the query sequences of the motif. An example of a result page is shown in Figure 2, using the sample DNA sequences provided on the server as the input (see detailed instructions in Supplementary Tutorial S2). The full information can be accessed through the job ID 2014012183209f.

For some applications, further analyses of the identified motifs may be needed. To facilitate this, the server provides three such analysis functionalities: motif scanning, motif comparison and clustering, and motif co-occurrence analyses, which can be directly applied to the predicted motifs on a result page, summarized as follows.

### Motif scanning in provided sequences for all instances of a query motif

We have implemented an algorithm, called BBS as part of the BoBro program, to scan for all instances of a specified motif in given sequences based on a global *P*-value estimation (15), which provides a reliable measure for the statistical significance of the scanned motif instances. This *P*-value can be used to derive an optimal similarity cut-off used for motif scanning on a statistically sound basis, which can effectively reduce the false-positive prediction rates compared to the existing prediction tools as we have demonstrated (15).

#### Input and analysis

Two input files are required to run this function: (i) a set of to-be-scanned DNA sequences in the FASTA format, and (ii) a set of provided motifs, represented as either aligned motif instances (Supplementary Table S3), motif consensus (Supplementary Table S4) or motif count matrix (Supplementary Table S5), where a motif count matrix is a 4 × *L* matrix, with *L* being the motif length and 4 denoting the four nucleotide types, and each element of the matrix is the number of occurrences of the corresponding nucleotide in the relevant position in the aligned motif sequences. Supplementary Tutorial S4 shows an example of how to submit a motif scanning job.

#### Main and the result pages for motif scanning

The main page for motif scanning has four functional menus/buttons: (i) **Input query motifs** prompts the user to select the format for the input motifs and allows to upload the provided motifs defined above; (ii) **Input query sequences** allows the user to upload the to-be-scanned DNA sequences defined above; and (iii) **Submit job** is the same as in the previous subsection. All the identified instances for the given motifs can be accessed on a result page through the job ID, with each row representing one query motif containing the following information: motif logo, motif length, the *P*-value, the number of identified motif instances, the genomic location for each instance and a graphical mapping of all the instances to the corresponding query DNA sequences.

### Motif comparison and clustering

The server provides a capability, the BBC program, for motif comparisons through assessing the similarities among the identified motifs (15). When calculating the similarities among the motifs, the algorithm takes into consideration both the similarities among the motifs and the weak sequence-conservation signals from the flanking regions of the motifs (if available), which improves the prediction sensitivities based on our systematic assessment (15). Using this capability, a minimum-spanning-tree-based algorithm has been implemented to cluster the provided motifs into groups, each of which contains similar motifs. Specifically, two different similarity thresholds, *T_1_* and *T_2_* (*T_1_* > *T_2_*), are used in the clustering algorithm, giving rise to a highly reliable and a relatively reliable motif group, respectively. We define a pair of motifs as highly similar if their similarity score is no less than *T_1_*, and as relatively similar if the similarity is no less than *T_2_* and less than *T_1_*.

The default values of these two thresholds are *T_1_* = 0.8 and *T_2_* = 0.4, which represent the upper quartile and the median of all the similarity scores, respectively, among all pairs of motifs as documented in the RegulonDB database. We refer the reader to (15) for details.

#### Input and analysis

The input to this functionality is a set of motifs to be compared, in the same formats as the ones for the motif-scan function (Supplementary Tables S3–S5). This functionality also allows a user to enter the original DNA sequences of the motifs if so chosen. This is for the purpose of getting the flanking regions of these motifs. An example for submitting a job for this functionality is described in Supplementary Tutorial S5.

#### Main and the result pages for motif comparison and clustering

**Figure 3.**
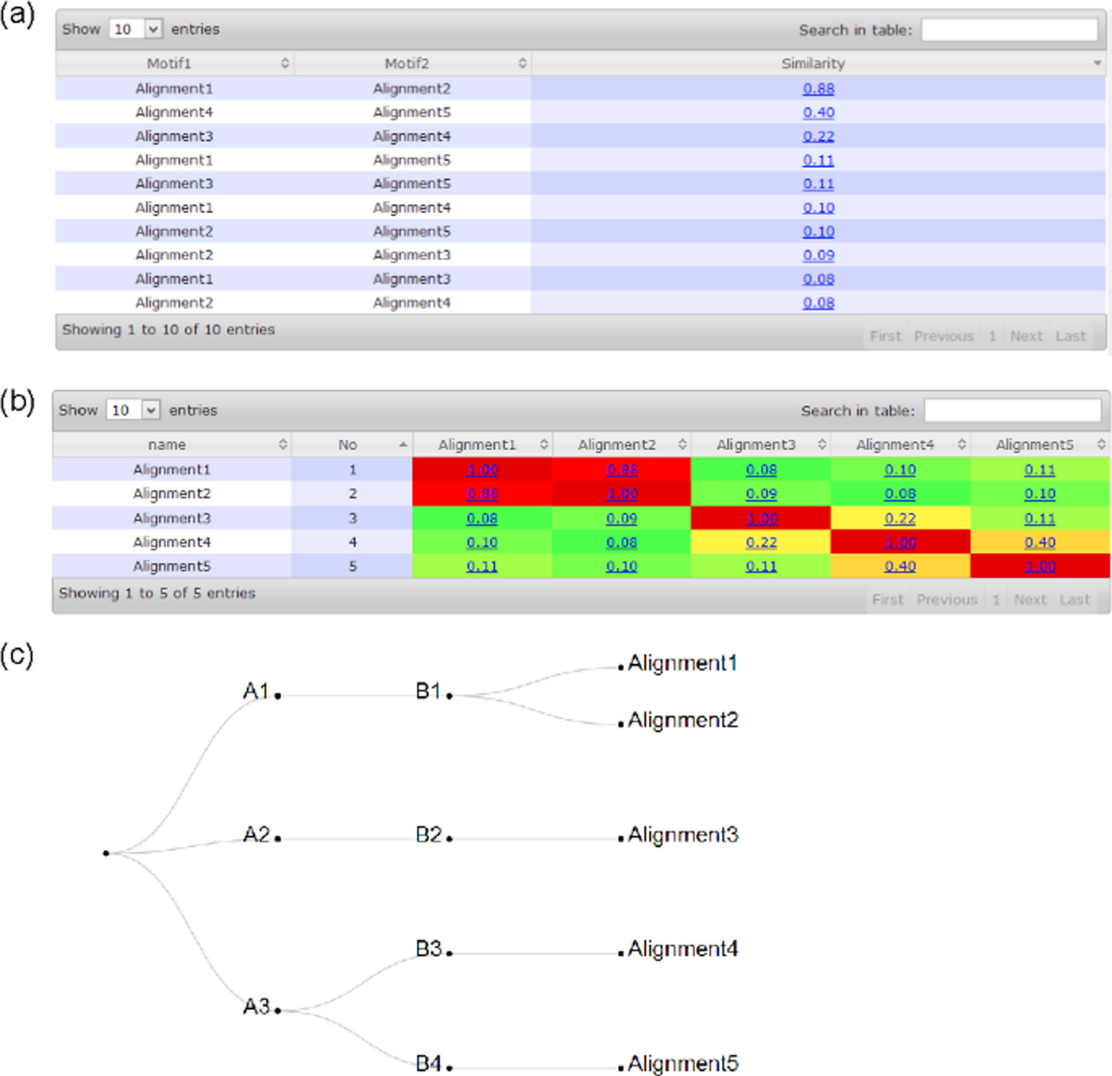
Result page of motif comparison and clustering using sample data set: (**a**) similarity score table, (**b**) similarity score heat-map and (**c**) clustering results.

The main page for this functionality has four menus/buttons, namely: (i) **Input query motifs** is the same as in the previous subsection, (ii) **Include host DNA sequences** prompts the user to include the DNA sequences in which the motifs are located if choosing to do so; (iii) **Set parameters** allows the user to adjust the above two motif-similarity thresholds, and (iv) **Submit job** is the same as in the previous subsection. The result page includes three parts: (i) a table containing the pairwise similarity score for each pair of given motifs, (ii) the same information as in (i) but represented as a heat-map, and (iii) clustering result represented in a hierarchical manner. Figure 3 shows a display of the computational results on the provided sample data as the input (see detailed instructions in Supplementary Tutorial S4). The detailed prediction information can be accessed through the job ID 20140313102801s.

### Motif co-occurrence analysis

**Figure 4.**
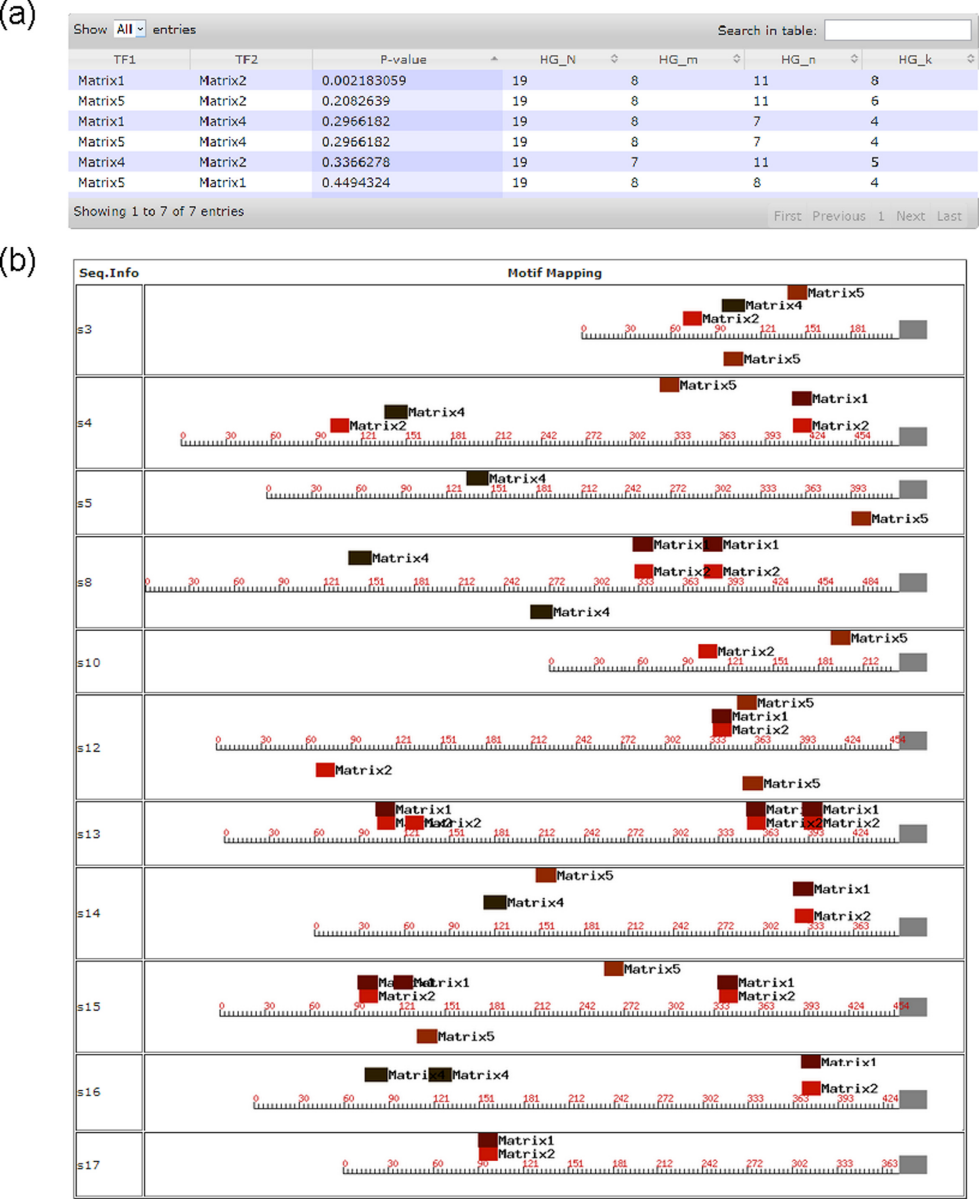
Result page of motif co-occurrence analysis using sample data set: (**a**) *P*-values for identified co-occurring motifs and (**b**) mapping of motifs to regulatory sequences.

Our server provides a capability for co-occurrence inference among the identified motifs, aiming to reveal joint regulation relationships by multiple TFs. A *P*-value is calculated for each pair of motifs to co-occur in the same regulatory regions as the probability of them sharing at least *k* regulatory motifs, modeled using a hyper-geometric function, where *k* represents the number of regulatory regions containing both motifs (15). The calculation is done using the BBA subroutine in BoBro and the detailed information about the method is given in (15). This function is only available to the user as a follow-up analysis in the result pages of motif identification and motif scanning, hence no input is required. The calculated *P*-values for each pair of co-occurring motifs are shown in a sortable table and the graphical mapping of motif instances to the regulatory sequences are displayed along with all the genes aligned on the right. See Figure 4 for a detailed display of the computing results on the sample datasets as the input (see detailed instructions in Supplementary Tutorial S5). The complete prediction information can be accessed through the job ID 20140313102638c.

### Motif identification and analyses: an application example

We use the following example to illustrate how the server can be used to solve a motif-finding problem in a prokaryotic genome. The tricarboxylic acid cycle (TCA cycle) consists of a series of oxidative reactions for energy (ATP) generation from carbohydrates, amino acids and lipids. The TCA cycle pathway consists of 28 enzymes in *E. coli* K-12, which are encoded by 28 genes covered by 17 operons according to the DOOR database. They are known to be transcriptionally co-regulated by three global TFs: CRP (cAMP receptor protein), FNR (fumarate and nitrate **r**eduction) and ArcA. See details in Supplementary Table S6 (33). The goal here is to show how the server can be used to predict the conserved *cis*-regulatory motifs of all the operons encoding this pathway using the following steps:

Step 1: Go to the front page of DMINDA and click on **Motif finding**. A new page for this functionality will pop out with the following menus: (i) **Input query sequences**, (ii) **Include control sequences**, (iii) **Set parameters** and (iv) **Submit job**.

Step 2: To prepare the promoter sequences for the relevant operons using the DOOR database, click on **Select from DOOR** under **Input query sequences**. Search for ‘NC_000913’ or ‘*E. coli* K-12 MG1655’ in the organism table. Click on ‘NC_000913’, and a table of operons for this genome will be shown along with a button **Get promoters.** To find all the operons containing genes in TCA cycle, search for each gene name, e.g. ‘aceE’, in the operon table and select each correct one by clicking on the box in the first column of the corresponding row. After all the operons containing TCA cycle genes are selected, click on **Get promoters** to get the corresponding promoters, which uploads the promoter sequences into the input box.

Step 3: One can include the entire genome of *E. coli* K-12 as a control for statistical significance estimation by clicking on **Select from DOOR** under **Include control sequences** and following similar steps to descriptions in the 'Motif identification and evaluation' section.

Step 4: One can now set the three selectable parameters of the current functionality: the number, the minimal and maximal lengths of to-be-identified motifs to 10, 14 and 16, respectively, through clicking on **Set parameters**. These numbers are so selected if one wants to find up to 10 distinct motifs and the sequence lengths of the predicted motifs are between 14 and 16, knowing that the motif lengths of FNR, ArcA and CRP are 14, 15 and 16, respectively, according to RegulonDB.

Step 5: Now one can click on **Submit** under **Submit job** to submit the prediction job. Here the user has the option to enter an email address for results retrieval or not.

For this example, DMINDA can finish motif finding within 238 s wall clock time, and the prediction results can be retrieved by entering the job ID 20140120153137f into the searching box on our server. According to the RegulonDB database, these promoter sequences contain 17, 5 and 3 experimentally validated binding sites for the three regulators ArcA, FNR and CRP, respectively. The overlaps between our prediction and the data of RegulonDB are 11, 3 and 2, respectively, with the details shown in Supplementary Table S7. The reason that the server did not identify all the experimentally verified motifs, we believe, is due to the relatively low sequence similarities between the missing motifs and the rest. It is worth noting that the server identified 18 motif instances for eight other TFs besides above three, indicating that additional regulatory mechanisms for the transcription of the TCA cycle genes may be used (Supplementary Table S7).

## IMPLEMENTATION

DMINDA is implemented as a web portal server with a multi-layer architecture. The representation and the logic layers are implemented using Web 2.0 technologies (HTML5, CSS3 and Javascript language along with jQuery library) and PHP server side scripting language. All data are stored in an optimized MySQL relational database. The web server runs on a Red Hat Enterprise Linux 6 box (16 Intel Xeon CPUs with 2.4 GHz and 16GB memory), and automated operon prediction pipeline runs on the computing cluster server with 150 computing nodes (two Intel Xeon CPUs with 3.06 GHz and 2.5GB memory per node, respectively).

## CONCLUDING REMARK

Motif identification along with the associated analyses is an important problem to the elucidation of transcriptional regulation mechanisms. While numerous algorithms and a number of web servers have been developed, the problem remains largely unsolved. Here we present a new web server based on a suite of algorithms that we previously developed and thoroughly tested on large data sets of both prokaryotic and human sequences with superior performances to the state-of-the-art motif-finding and analysis servers. We expect that this new server will prove to be a powerful motif-finding and analyses tool for comparative genome analyses, particularly for prokaryotic promoter analyses, complementary to the existing ones. We expect that as the next-generation sequencing data for TF binding become widely available for a large number of organisms, we will include such data to make our motif prediction more reliable.

## SUPPLEMENTARY DATA

Supplementary Data are available at NAR Online.

Supplementary Data
