# Supplementary material for: DMINDA: an integrated web server for DNA motif identification and analyses
Source: Nucleic Acids Res. 2014 Apr 21;42(Web Server issue):W12–9. doi: 10.1093/nar/gku315 (PMC4086085; doi:10.1093/nar/gku315)
Supplement: Supplementary Data [file supp_42_W1_W12__index.html]

Supplementary Data 

# DMINDA: an integrated web server for DNA motif identification and analyses

## Supplementary Data

**Files in this Data Supplement:**

- SUPPLEMENTARY DATA
