# Supplementary material for: DMINDA: an integrated web server for DNA motif identification and analyses
Source: Nucleic Acids Res. 2014 Apr 21;42(Web Server issue):W12–9. doi: 10.1093/nar/gku315 (PMC4086085; doi:10.1093/nar/gku315)
Supplement: Supplementary Data [file supp_gku315_nar-00155-web-b-2014-File006.pdf]

SUPPLEMENTARY DATA

Method S1: How do you de-novo identify motifs?

The de-novo motif finding program, **BOBRO**, is published on NAR, 2011.

Guojun Li, Bingqiang Liu, Qin Ma, Ying Xu, *A new framework for identifying cis-regulatory motifs in prokaryotes*, **Nucleic Acids Res.**2011 Apr; 39(7):e42

BOBRO is an algorithm for *cis*-regulatory motifs prediction in promoter sequences. The algorithm is based on two key ideas: (i) reliably assessing the possibility for each position in a given promoter to be the (approximate) start of a conserved sequence motif though a highly effective method; and (ii) reliably recognizing actual motifs from the accidental ones based on the concept of 'motif closure'. These two key ideas are embedded in a classical framework for motif finding through finding cliques in a graph but have made this framework substantially more sensitive as well as more selective in motif finding in a very noisy background. BOBRO substantially improves the prediction accuracy and extends the scope of applicability of the existing programs. The experiments on the promoter sets from E. coli K12 genome shows that the performance coefficient was improved from 29% to 41% by our program compared to the best among other six state-of-the-art prediction tools. The performance also consistently improved by substantial margins on another kind of large-scale data sets of orthologous promoters across multiple genomes. The power of BOBRO in dealing with noisy data was further demonstrated through identification of the motifs of the global transcriptional regulators by running it over 2390 promoter sequences of *Escherichia coli* K12. The related data sets and results can be found at: https://code.google.com/p/bobro/.

Tutorial S1: General introduction

Our web server is an integrated DNA motif analyses suite, whose infrastructure is shown on Figure 1 in main text. The users can kick off with motif finding by push the rectangle at the left-upper corner, which can lead them to the submit page. If the users are working on any sequenced prokaryotic species, click the DOOR2 logo, then they can select their query genome and operons, our server will prepare the promoter sequences automatically for the following-up motif analyses. The largest dark-green rectangle indicates our de-novo motif finding function, which is also the most important and common function in motif analysis. Its results are the basis of the advanced motif analyses: motif refinement, motif comparison and clustering; and motif occurrence. Each of the advanced functions is shown in the most right part, and the users can

access the specific functional analysis page by clicking the corresponding logos. The motif database cylinder links a collection of annotated motifs resources for both prokaryotic and eukaryotic species, in case the users want to start with some documented motifs for the advanced analyses functions.

Alternatively, the users can click the submit button on the navigation bar (see Fig. S1a) to access the job submission page, where they can upload their data to our server respect to a specific function. And our server supports the searching engine (Fig. S1b) when the users go back with a specific job ID, which can link them to the results of the job.

a.

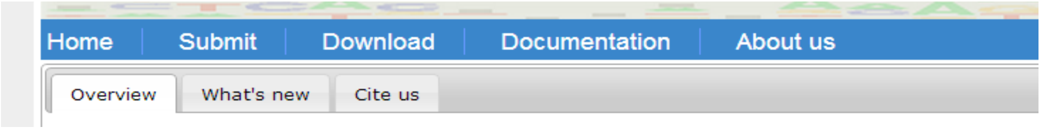

c.

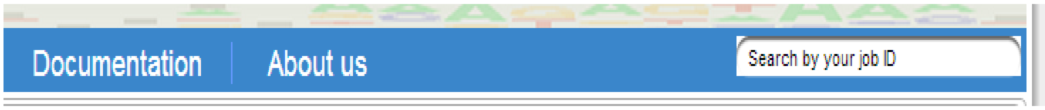

Figure S1: General introduction of the motif web server. (a) The navigator of the web server indicating Home Page, Submit analysis job, Download source code, Documentation, and About us; and (b) The search engine for users to find their submit work by a specific job ID, such as 2013121983904f.

Tutorial S2: How to submit a job for *De-novo* motif finding

The default page for submitting a job is designed for de-novo motif finding (see Fig. S2a). Totally, there are up to four steps to complete a job submission.

Step 1: Input query sequences: The requirement of sequence format can be found in FAQ 4. The users have three ways to upload the sequence: (i) paste the sequences in the corresponding box, see an example by selecting “sample”; (ii) let DOOR2 prepare the promoter sequences if they focus on bacteria; and (iii) upload a local file containing the query sequence. Please see the details of how to submit sequence using DOOR2 database in Tutorial S3.

Step 2: Include control sequences. It is optional and allow the user to include a set of background sequences as control, see details in FAQ 12. We can further evaluate the predicted motifs besides their P-values using formula (1) in FAQ 10. The format and submission requirement of background sequence is same to above query sequence.

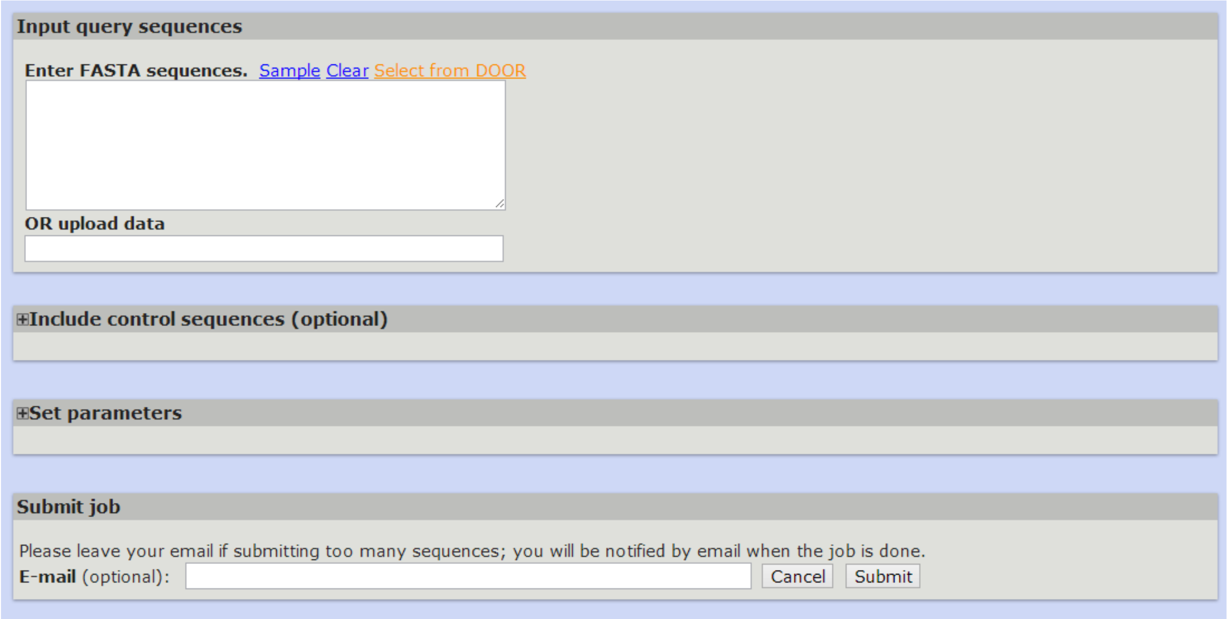

Figure S2: Submitting a job for motif finding. The default submitting page is for de-novo motif finding, include up to four steps: Input query sequence, Include control sequences, Set parameters, and Submit job.

Step 3: Set parameters. This one is optional.

Step 4: Submit job. Before selecting "Submit" button, the users can leave their emails, which will be contacted when the job is done. However this action is optional.

Specifically, to get the results shown in Figure 2, the users can just push the "sample" buttons in Step 1&2 and directly submit job in Step 4, without adjusting parameters in Step 3.

Tutorial S3: How to submit sequence using DOOR2 database

Whenever the users select a DOOR2 database logo, they will see Fig. S3a. Take *E. coli* K12 as an example. Firstly, type "NC_000913" in the searching bar at the right-upper corner, and you will get Fig. S3b. Select "NC_000913(C)" and then select the operons you are interested in, say the first 15 operons in the following page, and the select "Get promoters" in Fig. S3c. Then the users will be linked to the default submit page with your promoters pasted in the corresponding box, see Fig. S3d. And the users can go ahead to submit jobs, see details in Tutorial S2.

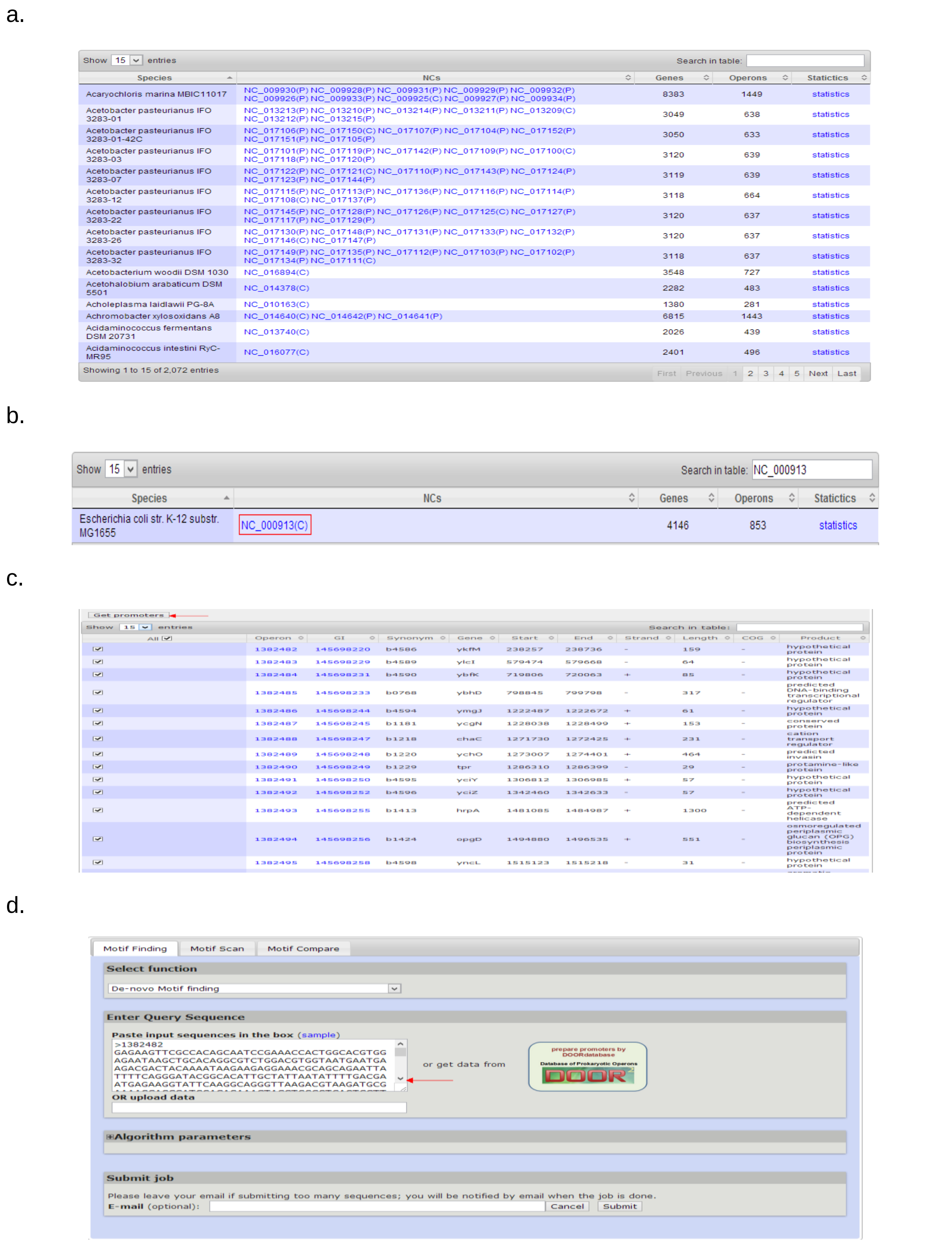

Figure S3. The procedure of how to prepare promoter sequences by DOOR database.

Tutorial S4: How to submit a job for motif scanning (Fig. S4)

Similar to *de-novo* motif finding submission page, there are four steps for submitting a motif scanning job:

Step 1: Input query motifs. The user is required to input motifs in selected motif format. Three kinds of format are accepted by our server, see details in FAQ 5. And the users can submit background sequences, if have, see details in FAQ 11-12.

Step 2: Input query sequences. In this step, the users is required to submit the to-be-scanned DNA sequences in FASTA format.

Step 3: same to the step 4 in Tutorial S2.

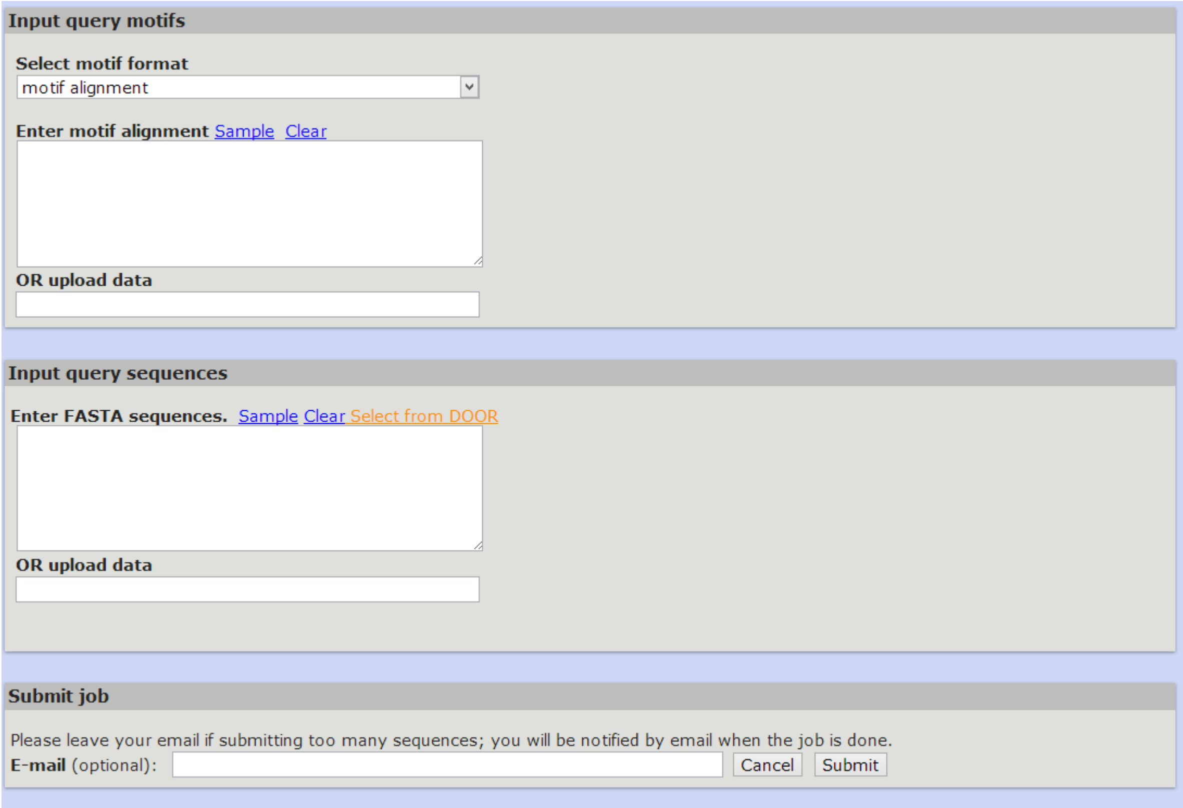

Figure S4: submitting a job for motif scanning.

Specifically, to get the results shown in Figure 3, the users can just push the “sample” buttons in Step 1&2 and directly submit job in Step 3.

Tutorial S5: How to submit a job for motif comparison and clustering, see Fig. S5.

Similar to Tutorial S2 and S4, there are four steps to submit a job for motif comparison and clustering.

Step 1: Input query motifs. In this step, the users are only allowed to submit the motifs in selected format.

Step 2: Input host DNA sequences. If the users can provide the origin sequences for the query motifs, our method can improve the motif comparison performance by considering the weak conserved signals of motifs' flanking regions, see details in FAQ 13. However this is optional and the default option is "no".

Step 3: same to the step 3 in Tutorial S2.

Step 4: same to the step 4 in Tutorial S2.

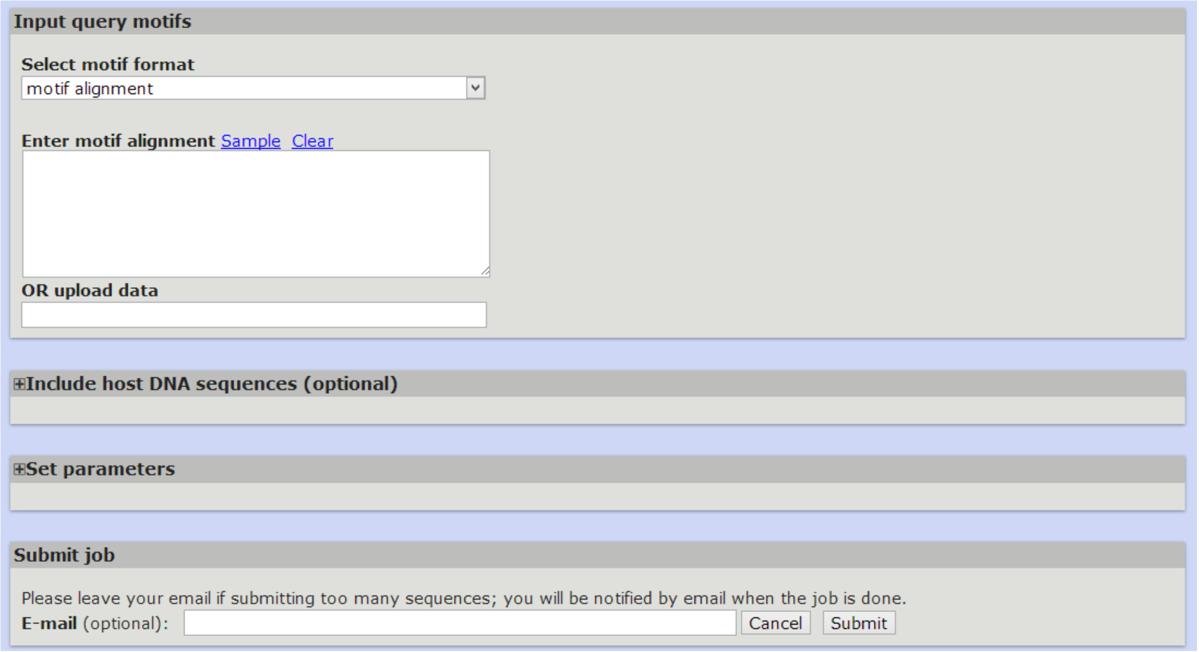

Figure S5: submitting a job for motif comparison and clustering.

Specifically, to get the results shown in Figure 4, the users can just push the "sample" button in Step 1 and directly submit job in Step 4, skipping the Steps 2&3.

Table S1. Collected DNA motif databases in public domain

Database	Class	Species	Reference
ATCOECIS	Eukaryotes	Arabidopsis	(1)
CollecTF	Prokaryotes	Bacteria	(2)

DBTBS	Prokaryotes	Bacillus subtilis	(3)
JASPAR	Eukaryotes	Eukaryotes	(4)
Paper	Eukaryotes	Yeast	(5)
MacIsaac	Eukaryotes	Yeast	(6)
MAPPER2	Eukaryotes	human, mouse, and D.melanogaster	(7)
Paper	Eukaryotes	Human and mouse	(8)
Paper	Eukaryotes	Human (CTCF)	(9)
Paper	Eukaryotes	Mammalian (ETS-family)	(10)
Paper	Eukaryotes	Human and mouse (SELEX)	(11)
Paper	Eukaryotes	Mouse (Embryonic stem)	(12)
Paper	Eukaryotes	Mouse (homeodomain)	(13)
Paper	Eukaryotes	Drosophila	(14)
PLACE	Eukaryotes	Plants	(15)
FlyFactorSurvey	Eukaryotes	Drosophila	(16)
RegPrecise 3.0	Prokaryotes	Bacteria	(17)
RegTransBase	Prokaryotes	Prokaryotes	(18)
RegulonDB	Prokaryotes	E. coli	(19)
PRODORIC	Prokaryotes	Prokaryotes	(20)
UNIPROBE	Both	Vibrio harveyi, Plasmodium falciparum, Cryptosporidium parvum, Saccharomyces cerevisiae, Caenorhabditis elegans,mouse, and human	(21)

Table S2. The actual computational time of samples and some large-scale jobs on DMINDA. Note: The number of output motifs should be less than 100, otherwise they will be too slow to be displayed.

	BoBro	BBS	BBC	BBA
**Sample data**	JobID: 20140316135117f	JobID:	JobID:	Input: 8 motifs and

	Input: 19 promoters<br>Output: 8 motifs<br>Time: 120s	20140316133439s<br>Input: 5 motifs and 19 promoters<br>Time: 50s	20140316133454c<br>Input: 5 motifs<br>Time: 9s	19 promoters<br>Time:6s
**TCA cycle example**	JobID: 20140120153137f<br>Input: 17 promoters<br>Output: 10 motifs<br>Time: 238s	JobID: 2014031691048s<br>Input: 10 motifs and 17 promoters<br>Time: 74s	JobID: 2014031691441c<br>Input: 10 motifs and 17 promoters<br>Time:16s	Input: 17 promoters and 10 motifs<br>Time: 8s
**Bacterial whole genome (NC_012034)**	JobID: 20140316125905f<br>Input: 1,272 promoters<br>Output: 80 motifs<br>Time: 6,908s	Job ID: 20140316151804s<br>Input: 80 motifs and 1,272 promoters<br>Time: 448s	JobID: 20140316151926c<br>Input: 80 motifs and 1,272 promoters<br>Time: 20s	Input: 80 motifs and 1272 promoters<br>Time: 527s
**Human genome**	N/A	JobID: 20140316101840s<br>Input: 5 motifs and 20,044 promoters<br>Time: 447s	JobID: 20140316103154c<br>Input: 5 motifs and 20,044 promoters<br>Time: 13s	Input: 5 motifs and 20,044 promoters<br>Time: 4s
**Limit of to-be-shown motifs**	100	100	100	100

Table S3. One example of aligned motif instances.

>alignment														
A	A	C	A	T	T	T	A	G	T	T	A	A	C	C
T	A	A	A	A	A	T	T	G	T	T	A	A	C	A
A	A	A	A	C	T	T	G	A	T	T	A	A	C	A
A	A	C	A	T	T	T	A	G	T	T	A	A	C	T
A	A	C	A	A	T	T	A	T	T	T	A	A	C	A
T	A	A	T	T	A	T	T	A	T	T	A	A	C	C
A	A	A	A	T	A	T	A	A	T	G	A	A	C	A

Table S4. Three examples of motif consensus.

>Concensus1
CTAGGSMWGRAASC
>Concensus2
TAGMSMWGRAASC
>Concensus3
NAGCTGAAWYGTTTHDRTCCCA

Where,

W = A or T
S = C or G
R = A or G
Y = C or T
K = G or T
M = A or C
B = C, G, or T (not A)
D = A, G, or T (not C)
H = A, C, or T (not G)
V = A, C, or G (not T)
N = A, C, G, or T

Table S5. One example of motif count matrix.

>matrix									
A	40	47	23	42	23	33	12	23	40
G	5	6	8	9	5	15	0	13	26
C	7	5	30	7	14	1	5	14	0
T	23	17	14	17	33	26	58	25	9

Table S6. The 28 genes included in the TCA cycle pathway of *E. coli K-12*, along with the documented TFBSs covered by our prepared promoters regarding RegulonDB. The names of corresponding TFs are listed in the fourth column (with the TFBSs number following in the brackets). Note: we only consider the TFs regulating over three operons in our analysis.

Locus ID	Gene name	Operon ID	Transcription Factors
**b0114**	aceE	3024	NsrR(1)
**b0115**	aceF	3024	
**b0116**	lpd	1382546	ArcA(2), CRP(1), Fis(1)
**b0118**	acnB	1382548	ArcA(7), Fis(3)
**b0615**	citF	3125	ArcA(1), CRP(1), DpiA(3), FNR(1), NarL(1)
**b0616**	citE	3125	
**b0617**	citD	3125	
**b0720**	gltA	1382702	ArcA(1)
**b0721**	sdhC	3144	ArcA(2), Fur(1)
**b0722**	sdhD	3144	
**b0723**	sdhA	3144	
**b0724**	sdhB	3144	
**b0726**	sucA	3145	
**b0727**	sucB	3145	
**b0728**	sucC	3146	
**b0729**	sucD	3146	
**b0771**	ybhJ	1383778	
**b1136**	icd	1382814	ArcA(2), Cra(1)
**b1276**	acnA	1382860	ArcA(1), CRP(1), FNR(1), MarA(1), Rob(1), SoxS(1)
**b1611**	fumC	1382955	Rob(1), SoxS(1)

**b1612**	fumA	1382956	CRP(1)
**b3236**	mdh	1383329	ArcA(1), CRP(1), Cra(1)
**b3403**	pck	1383352	
**b4122**	fumB	3797	FNR(1), NarL(3)
**b4151**	frdD	3804	DcuR(1), NarL(5)
**b4152**	frdC	3804	
**b4153**	frdB	3804	
**b4154**	frdA	3804	

Table S7. The information of predicted motif instances in the TCA cycle example, which can match documented TFBSs in RegulonDB. All the information are downloaded from the result of the job 20140120153137f, and a predicted motif instance is called matched to a TFBS if its genomic range is overlap with that of the TFBS.

Motif instance	Operon	start	end	TF	TFBS start	TFBS end	TFBS strand
**ATTAAATCAATTAA**	3125	651101	651114	ArcA	651107	651116	reverse
**TATATGTAGGTTAA**	3144	754131	754144	ArcA	754142	754156	forward
**TAATTGTAATGATTTT**	3144	754142	754157	ArcA	754147	754161	forward
**AAATTGTTAACAATTT**	1382546	127689	127704	ArcA	127683	127697	forward
**AAATTGTTAACAATTT**	1382546	127689	127704	ArcA	127692	127706	forward
**TAAATTTTGACTAA**	1382548	131346	131359	ArcA	131342	131356	forward
**TTGTAAACAGATTAAC**	1382548	131475	131490	ArcA	131464	131478	forward
**TTACAAATCATTAACA**	1382814	1194238	1194253	ArcA	1194226	1194240	forward
**TTACAAATCATTAACA**	1382814	1194238	1194253	ArcA	1194233	1194247	forward
**TGTTATCAAATCGTTA**	1382860	1333765	1333780	ArcA	1333764	1333778	forward
**CAAATTCTGCTTAA**	1383329	3382310	3382323	ArcA	3382302	3382316	reverse
**AAATTGTTAACAATTT**	1382546	127689	127704	CRP	127688	127709	forward
**TTGTAAACAGATTA**	1382548	131475	131488	CRP	131471	131492	forward
**TGTTATCAAATCGTTA**	1382860	1333765	1333780	CRP	1333754	1333775	forward
**AAACAAAACATTAACA**	3797	4346810	4346825	FNR	4346826	4346839	reverse
**TGTTATCAAATCGTTA**	1382860	1333765	1333780	FNR	1333758	133377	forward

						1	
**ATTGTAATGATTTT**	3144	754144	754157	Fur	754138	754156	forward
**CAACCCAAATTGAT**	1382860	1333753	1333766	MarA	1333744	1333763	forward
**GAACAAAAAATAGACC**	3797	4346770	4346785	NarL	4346756	4346771	reverse
**GAACAAAAAATAGACC**	3797	4346770	4346785	NarL	4346761	4346776	reverse
**GAACAAAAAATAGACC**	3797	4346770	4346785	NarL	4346778	4346793	reverse
**AAACAAAACATTAACA**	3797	4346810	4346825	NarL	4346824	4346839	reverse
**TAGTAATTAAATTAAT**	3804	4380438	4380453	NarL	4380431	4380446	reverse
**TAGTAATTAAATTAAT**	3804	4380438	4380453	NarL	4380439	4380454	reverse
**CAACCCAAATTGAT**	1382860	1333753	1333766	Rob	1333744	1333763	forward
**TAAAAGTTGCTTAA**	1382955	1684695	1684708	Rob	1684704	1684723	reverse
**CAACCCAAATTGAT**	1382860	1333753	1333766	SoxS	1333744	1333763	forward
**AAAGAAAAAATTAATC**	1382955	1684723	1684738	SoxS	1684705	1684724	reverse
**ATGTTGTTATCGATTT**	3125	651340	651355	DcuR	651350	651366	forward
**AAAAGGTTATCAGTTT**	3125	651300	651315	DpiA	651299	651321	reverse
**GTTGTTATCGATTT**	3125	651340	651353	DpiA	651341	651363	reverse
**AAATTGTTAACAATTT**	1382546	127689	127704	Fis	127681	127695	forward
**TAAATTTTGACTAA**	1382548	131346	131359	Fis	131340	131354	forward

REFERENCES

1. Vandepoele, K., Quimbaya, M., Casneuf, T., De Veylder, L. and Van de Peer, Y. (2009) Unraveling transcriptional control in Arabidopsis using cis-regulatory elements and coexpression networks. *Plant physiology*, **150**, 535-546.

2. Kilic, S., White, E.R., Sagitova, D.M., Cornish, J.P. and Erill, I. (2013) CollecTF: a database of experimentally validated transcription factor-binding sites in Bacteria. *Nucleic acids research*.
3. Sierro, N., Makita, Y., de Hoon, M. and Nakai, K. (2008) DBTBS: a database of transcriptional regulation in Bacillus subtilis containing upstream intergenic conservation information. *Nucleic acids research*, **36**, D93-96.
4. Mathelier, A., Zhao, X., Zhang, A.W., Parcy, F., Worsley-Hunt, R., Arenillas, D.J., Buchman, S., Chen, C.Y., Chou, A., Ienasescu, H. *et al.* (2013) JASPAR 2014: an extensively expanded and updated open-access database of transcription factor binding profiles. *Nucleic acids research*.
5. Gordan, R., Murphy, K.F., McCord, R.P., Zhu, C., Vedenko, A. and Bulyk, M.L. (2011) Curated collection of yeast transcription factor DNA binding specificity data reveals novel structural and gene regulatory insights. *Genome biology*, **12**, R125.
6. MacIsaac, K.D., Wang, T., Gordon, D.B., Gifford, D.K., Stormo, G.D. and Fraenkel, E. (2006) An improved map of conserved regulatory sites for Saccharomyces cerevisiae. *BMC bioinformatics*, **7**, 113.
7. Marinescu, V.D., Kohane, I.S. and Riva, A. (2005) The MAPPER database: a multi-genome catalog of putative transcription factor binding sites. *Nucleic acids research*, **33**, D91-97.
8. Wei, G.H., Badis, G., Berger, M.F., Kivioja, T., Palin, K., Enge, M., Bonke, M., Jolma, A., Varjosalo, M., Gehrke, A.R. *et al.* (2010) Genome-wide analysis of ETS-family DNA-binding in vitro and in vivo. *The EMBO journal*, **29**, 2147-2160.
9. Xie, X., Mikkelsen, T.S., Gnirke, A., Lindblad-Toh, K., Kellis, M. and Lander, E.S. (2007) Systematic discovery of regulatory motifs in conserved regions of the human genome, including thousands of CTCF insulator sites. *Proceedings of the National Academy of Sciences of the United States of America*, **104**, 7145-7150.
10. Hallikas, O., Palin, K., Sinjushina, N., Rautiainen, R., Partanen, J., Ukkonen, E. and Taipale, J. (2006) Genome-wide prediction of mammalian enhancers based on analysis of transcription-factor binding affinity. *Cell*, **124**, 47-59.
11. Jolma, A., Kivioja, T., Toivonen, J., Cheng, L., Wei, G., Enge, M., Taipale, M., Vaquerizas, J.M., Yan, J., Sillanpaa, M.J. *et al.* (2010) Multiplexed massively parallel SELEX for characterization of human transcription factor binding specificities. *Genome research*, **20**, 861-873.
12. Chen, X., Xu, H., Yuan, P., Fang, F., Huss, M., Vega, V.B., Wong, E., Orlov, Y.L., Zhang, W., Jiang, J. *et al.* (2008) Integration of external signaling pathways with the core transcriptional network in embryonic stem cells. *Cell*, **133**, 1106-1117.
13. Berger, M.F., Badis, G., Gehrke, A.R., Talukder, S., Philippakis, A.A., Pena-Castillo, L., Alleyne, T.M., Mnaimneh, S., Botvinnik, O.B., Chan, E.T. *et al.* (2008) Variation in homeodomain DNA binding revealed by high-resolution analysis of sequence preferences. *Cell*, **133**, 1266-1276.
14. Kulakovskiy, I.V., Favorov, A.V. and Makeev, V.J. (2009) Motif discovery and motif finding from genome-mapped DNase footprint data. *Bioinformatics*, **25**, 2318-2325.
15. Higo, K., Ugawa, Y., Iwamoto, M. and Korenaga, T. (1999) Plant cis-acting regulatory DNA elements (PLACE) database: 1999. *Nucleic acids research*, **27**, 297-300.
16. Zhu, L.J., Christensen, R.G., Kazemian, M., Hull, C.J., Enuameh, M.S., Basciotta, M.D., Brasefield, J.A., Zhu, C., Asriyan, Y., Lapointe, D.S. *et al.* (2011) FlyFactorSurvey: a database of Drosophila transcription factor binding specificities determined using the bacterial one-hybrid system. *Nucleic acids research*, **39**, D111-117.
17. Novichkov, P.S., Laikova, O.N., Novichkova, E.S., Gelfand, M.S., Arkin, A.P., Dubchak, I. and Rodionov, D.A. (2010) RegPrecise: a database of curated genomic inferences of

transcriptional regulatory interactions in prokaryotes. *Nucleic acids research*, **38**, D111-118.
18. Cipriano, M.J., Novichkov, P.N., Kazakov, A.E., Rodionov, D.A., Arkin, A.P., Gelfand, M.S. and Dubchak, I. (2013) RegTransBase--a database of regulatory sequences and interactions based on literature: a resource for investigating transcriptional regulation in prokaryotes. *BMC genomics*, **14**, 213.
19. Salgado, H., Peralta-Gil, M., Gama-Castro, S., Santos-Zavaleta, A., Muniz-Rascado, L., Garcia-Sotelo, J.S., Weiss, V., Solano-Lira, H., Martinez-Flores, I., Medina-Rivera, A. *et al.* (2013) RegulonDB v8.0: omics data sets, evolutionary conservation, regulatory phrases, cross-validated gold standards and more. *Nucleic acids research*, **41**, D203-213.
20. Munch, R., Hiller, K., Barg, H., Heldt, D., Linz, S., Wingender, E. and Jahn, D. (2003) PRODORIC: prokaryotic database of gene regulation. *Nucleic acids research*, **31**, 266-269.
21. Robasky, K. and Bulyk, M.L. (2011) UniPROBE, update 2011: expanded content and search tools in the online database of protein-binding microarray data on protein-DNA interactions. *Nucleic acids research*, **39**, D124-128.
